# Knowledge about cervical cancer risk factors and human papilloma virus vaccine among Saudi women of childbearing age: A community-based cross-sectional study from Saudi Arabia

**DOI:** 10.1016/j.jvacx.2023.100361

**Published:** 2023-07-28

**Authors:** Abdulrahim Gari, Manar A. Ghazzawi, Shahad A. Ghazzawi, Shahd M. Alharthi, Elaf A. Yanksar, Rawan M. Almontashri, Raghad Batarfi, Lina I. Kinkar, Saeed Baradwan

**Affiliations:** a Department of Obstetrics and Gynecology, Faculty of Medicine, Umm Al-Qura University, Makkah, Saudi Arabia; b Department of Obstetrics and Gynecology, King Faisal Specialist Hospital and Research Center, Jeddah, Saudi Arabia; c Faculty of Medicine, Umm Al-Qura University, Makkah, Saudi Arabia; d Department of Neonatologist Nursing, Hera General Hospital, Makkah Healthcare Cluster, Makkah, Saudi Arabia

**Keywords:** Knowledge, Awareness and practice, Human Papilloma Virus Vaccine, HPV

## Abstract

**Objective:**

To examine the knowledge of cervical cancer risk factors and human papilloma virus (HPV) vaccine among Saudi women of childbearing age.

**Methods:**

An anonymous, survey-based, cross-sectional study was conducted from November 2022 to March 2023.

**Results:**

Overall, 422 participants were included in the current study. Most participants were within the age group of 15–25 years old (42.9%), single (47.9%), and educated with a bachelor's degree (70%). Out of a total of 14 points, the average knowledge score for all participants was 7.3 ± 2.31 (range: 2–14). More than three-quarters of the surveyed participants correctly identified the following risk factors for cervical cancer: multiple sexual partners (78.2%), having weakened immunity (82.7%), infection with HPV (82.9%), and positive family history of cervical cancer (88.9%). Concerning HPV vaccine, 153 (36.3%) participants heard about HPV vaccine and only 20 (4.4%) of them were vaccinated. Only 128 (30.3%) participants stated correctly that 9–13 years old is the best age to start HPV vaccine, whereas 51 (12.1%) participants correctly stated the number of HPV vaccine doses to be three over six months. Overall, 167 (39.6%) participants declined to receive the HPV vaccine. The three most frequently reported reasons included not hearing about HPV vaccine (35.3%), fear from HPV-related side effects (30.5%), and apprehension from HPV vaccine injection (16.2%). Among several socio-demographic characteristics, occupation was statistically significantly associated with knowledge score (p < 0.001), with students in health specialties tended to have the highest knowledge score compared with others.

**Conclusion:**

Most participants displayed good knowledge about cervical cancer risk factors, but not about HPV vaccine. Very alarmingly, less than 5% of the participants received HPV vaccine and close to 40% of them declined to receive the HPV vaccine. Mechanisms to increase public awareness about HPV vaccine and its acceptance by women are recommended.

## Introduction

Cervical cancer is a common gynecologic malignancy worldwide, and its burden is specifically higher in countries with low- and middle-income countries [1]. Human papilloma virus (HPV) is a frequent cause of cervical cancer [2], [3]. To a larger extent, cervical cancer is a preventable disease via various HPV-based screening methods (for example, pap smear) and HPV vaccine intervention methods [4]. Most cervical premalignant changes evolve slowly over time, so potential cancer lesions can usually be thwarted if a woman is routinely screened [4], [5]. Additionally, HPV vaccines can substantially reduce the occurrence and severity of HPV-related diseases [6]. For example, one randomized, double-blinded, controlled trial depicted that HPV vaccine can extensively reduce the rates of various HPV infection types (for example, 16 and 18) and high-grade cervical lesions by 90% and 85%, respectively [7]. The World Health Organization (WHO) advises that individuals (inclusive of both genders) aged 9–14 years should receive a two-dose administration of the HPV vaccine [8]. Furthermore, females aged 13–26 years should receive a third dose of the HPV vaccine [8]. Indeed, countries that exercise solid HPV vaccine programs have observed a remarkable decline in the frequency of HPV-related infections [9].

In Saudi Arabia, cervical cancer is not uncommon and ranks among the top 10 most frequently reported malignancies [10]. Poor awareness about HPV vaccine in the developing Arabic countries is a key contributing factor to the high morbidity and mortality of cervical cancer [11]. In 2010, the Saudi Arabian Food and Drug Administration (FDA) has greenlighted two HPV vaccines (Gardasil and Cervarix) for female individuals aged 11–26 years, and it is offered cost-free by the government. Nevertheless, the uptake rate of HPV vaccine continues to be negligible owing to a multitude of reasons, most outstandingly is the dearth of awareness of HPV vaccine importance, efficacy, and safety. The matter is further compounded by HPV vaccine hesitancy [12].

Studies that concentrate on gauging the knowledge, attitude, and practice for cervical cancer prevention via HPV vaccine among the community are of pivotal importance. Such studies play central roles in assessing the public’s level of understanding of signs, symptoms, risk factors, advantages of early diagnosis, among others. Overall, these studies allow to identify strengths that are strengthened and to facilitate the detection of weaknesses that are rationally corrected through targeted policies and other remedies. Several lines of investigations endeavored to examine the knowledge and perceived attitudes toward HPV vaccine in Saudi Arabia across various cities and regions [12], [13], [14], [15], [16], [17], [18], [19], [20], [21], [22]. Nonetheless, to the best of our knowledge, no such study has explored the perceptions of Saudi women of childbearing age. Hence, such investigation is greatly warranted to fill up the research lacunae and enrich the existing body of literature on the topic of HPV vaccine in Saudi Arabia.

Therefore, the purpose of our study is to evaluate the extent of knowledge, awareness, and practice for HPV vaccine among Saudi women of childbearing age. Additionally, this investigation aims to scrutinize the relationship between various socio-demographic characteristics of the studied participants and their knowledge regarding cervical cancer prevention.

## 3 Materials and methods

The present investigation employed an anonymous, self-rating, survey-based, cross-sectional study conducted from November 2022 to March 2023. The inclusion criteria consisted of all female individuals of childbearing age (9–52 years) living in Saudi Arabia with complete survey responses. The exclusion criteria comprised male individuals, female individuals of non-childbearing age (<9 and >52 years), and incomplete survey responses.

To examine the surveyed participants' knowledge about cervical cancer risk factors and HPV vaccine, an electronic survey was formulated using Google Forms, which was then administered online and anonymously. The survey was devised based on three published articles [20], [21], [23], which was then revised and translated into Arabic language by professional experts. Next, the survey was examined for validity and reliability. Validity was examined via pilot testing on 10 individuals to ensure language clearness and question comprehensibility [24]. Reliability was examined via Cronbach’s α coefficient test and a value of >0.7 reflected an acceptable internal reliability of the data [25]. Collectively, the survey covered two sections. The first section comprised baseline socio-demographic characteristics of the surveyed participants. The second section comprised knowledge of respondents about cervical cancer risk factors and HPV vaccine using yes/no (n=11) and multiple-choice (n=3) questions. These 14 questions were used to gauge the knowledge score about cervical cancer and HPV vaccine; the minimum and maximum points were 0 and 14, respectively.

According to the WorldoMeters, the estimated current population of Saudi Arabia is 35,734,581 individual. It is estimated that at least 15% of Saudi Arabian population are below 18 years old (nearly 5,500,000 individuals). Hence, a population size of nearly 30,000,000 individuals in Saudi Arabia, 5% margin of error, and 95% confidence interval, the needed sample size calculation is 384 participants (https://www.surveysystem.com/sscalc.htm). In order to compensate for potential data attrition, we amended the total sample size to 422 participants. The study group was determined by convenience sampling method.

Descriptive data were summarized as numbers and percentages. Numerical data were summarized as means ± standard deviations (SD) and ranges (minimum–maximum). Numerical parameters were examined using independent samples *t*-test or one-way ANOVA test. Categorical parameters were examined using chi-square test. All analyses were two-tailed and p < 0.05 indicated statistical significance. Data analysis was carried out through the Statistical Package for Social Sciences (SPSS) software for Mac, version 23. The study protocol was approved by the Institutional Review Board (IRB) vide Letter No. HAPO-02-K-012-2022-11-1227 dated 02/11 2022 from Umm Al-Qura University, Makkah, Saudi Arabia.

## 3 Results

Overall, 422 participants were included in the current study. Table 1 summarizes the socio-demographic characteristics of the participants. Most participants were within the age group of 15–25 years old (n=181, 42.9%), single (n=202, 47.9%), educated with a bachelor's degree (n=298, 70%), and had total monthly income of more than 10,000 SAR (n=189, 44.8%). About 160 (37.9%) participants were unemployed, 103 (24.4%) participants were non-healthcare employees, 67 (15.9%) participants were students in health specialties, 55 (13%) participants were students in non-health specialties, and 37 (8.8%) participants were healthcare employees. Of the 195 married participants, 117 (60%) of the participants’ husbands were bachelor’s degree holders and 167 of them (85.6%) were non-healthcare workers.

**Table 1:** The socio-demographic characteristics of the surveyed participants (n=422).

**Variable**	**Categories**	**Frequency**	**Percent**
**Age in years (n=422)**	Less than 15	3	0.7
	15–25	181	42.9
	26–35	136	32.2
	36–45	55	13
	More than 45	47	11.1
**Marital status (n=422)**	Single	202	47.9
	Married	195	46.2
	Divorced	13	3.1
	Separated	6	1.4
	Widowed	6	1.4
**Education (n=422)**	Not educated	1	0.2
	High school or less	83	19.7
	Bachelor's degree	298	70.6
	Postgraduate degree	40	9.5
**Occupation (n=422)**	Healthcare employee	37	8.8
	Non-healthcare employee	103	24.4
	Unemployed	160	37.9
	Student in health specialties	67	15.9
	Student in non-health specialties	55	13
**Husband’s education (n=195)**	High school or less	67	34.4
	Bachelor's degree	117	60
	Postgraduate degree	11	5.6
**Husband’s occupation (n=195)**	Healthcare worker	28	14.4
	Non-healthcare worker	167	85.6
**Total perceived family monthly income in SAR (n=422)**	Less than 5,000	76	18
	5,000–10,000	157	37.2
	More than 10,000	189	44.8

**Fig. 1:**
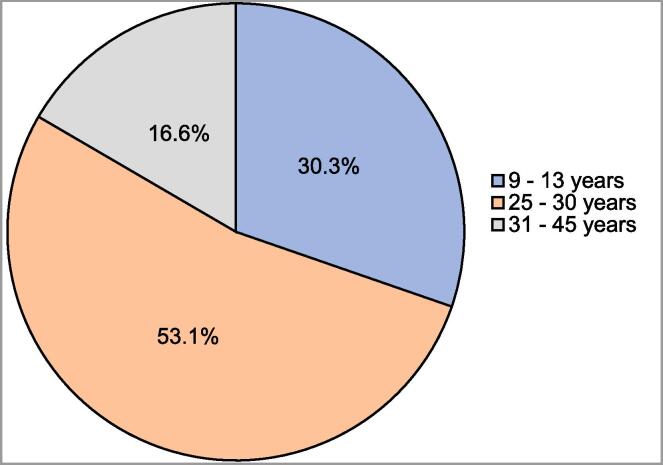
A pie chart depicting the responses of the surveyed participants to the question about what the best age is to start giving the HPV vaccine (n=422).

**Fig. 2:**
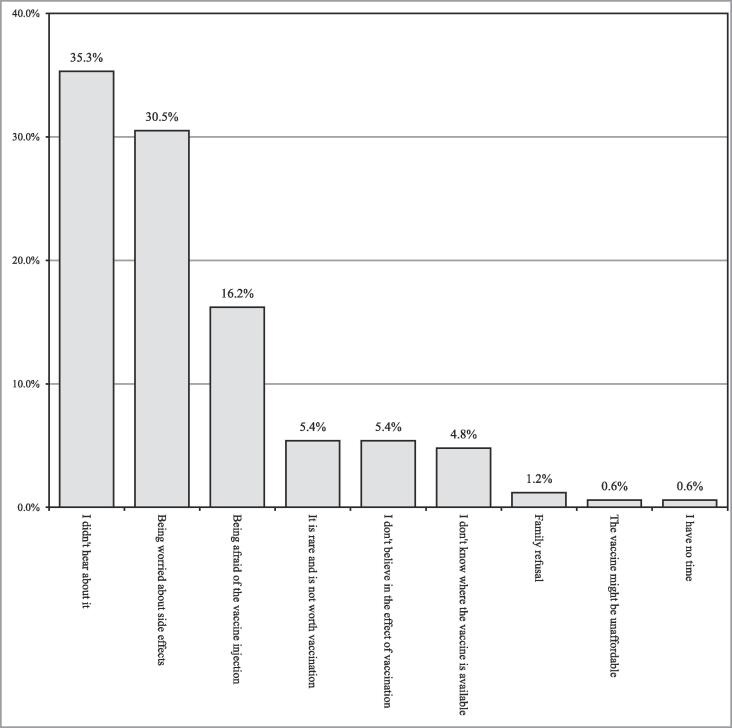
A bar graph depicting the responses of the surveyed participants to the reasons for refusal to receive HPV vaccine (n=167).

Table 2 summarizes the knowledge about cervical cancer risk factors and HPV vaccine among women of childbearing age (n=422). Out of a total of 14 points, the average knowledge score for all participants was 7.3 ± 2.31 (range: 2–14). Concerning knowledge about risk factors for cervical cancer, more than three-quarters of the surveyed participants correctly identified the following risk factors: multiple sexual partners (78.2%), having weakened immunity (82.7%), infection with HPV (82.9%), and positive family history of cervical cancer (88.9%). Close to half of the surveyed participants (49.3%) correctly identified early engagement in sex as a risk factor for cervical cancer. Only 61 (14.5%) participants correctly identified having many children as a risk factor for cervical cancer. Concerning HPV vaccine, 153 (36.3%) participants heard about HPV vaccine and only 20 (4.4%) of them were vaccinated. More than half of the participants (n=255, 60.4%) agreed to take HPV vaccine, whereas 208 (49.3%) participants claimed to know the purpose of receiving the HPV vaccine. Additionally, 128 (30.3%) participants stated correctly that 9–13 years old is the best age to start HPV vaccine (Fig. 1), whereas 51 (12.1%) participants correctly stated the number of HPV vaccine doses to be three over six months (Fig. 2).

**Table 2:** Knowledge about cervical cancer risk factors and HPV vaccine among women of childbearing age (n=422).

**Risk factors of cervical cancer**	**Yes, n (%)**	**No, n (%)**
Multiple sexual partners	330 (78.2)	92 (21.8)
Having many children	61 (14.5)	361 (85.5)
Starting to have early sex	208 (49.3)	214 (50.7)
Having a weakened immunity	349 (82.7)	73 (17.3)
Having history of sexually transmitted infection	338 (80.1)	84 (19.9)
Use of oral contraceptive pills	167 (39.6)	255 (60.4)
Smoking cigarette	270 (64)	152 (36)
Infection with human papilloma virus	350 (82.9)	72 (17.1)
Not using condom during sex	110 (26.1)	312 (73.9)
Family history of cervical cancer	375 (88.9)	47 (11.1)
**Have you ever heard about HPV vaccine?**	153 (36.3)	269 (63.7)
**Were you vaccinated for HPV?**	20 (4.7)	402 (95.3)
**Do you agree to take the human papilloma virus vaccine?**	255 (60.4)	167 (39.6)
**Do you know what the purpose is of taking the HPV vaccine?**	208 (49.3)	214 (50.7)

Fig. 2 summarizes all reasons for refusal to receive HPV vaccine. Overall, 167 (39.6%) participants declined to receive the HPV vaccine. The three most frequently reported reasons included not hearing about HPV vaccine (35.3%), fear from HPV-related side effects (30.5%), and apprehension from HPV vaccine injection (16.2%). Only marginal fractions of participants reported family refusal (1.2%), HPV vaccine unaffordability (0.6%), and lack of time (0.6%) as potential roots for refusal to receive the HPV vaccine.

Table 3 summarizes correlations between various socio-demographic characteristics of the surveyed participants and their knowledge regarding cervical cancer risk factors and HPV vaccine. Overall, no statistically significant differences were identified between age, marital status, education, husband’s occupation, husband’s education, and monthly income with knowledge score (p > 0.05). However, occupation was statistically significantly associated with knowledge score (p < 0.001), with students in health specialties tended to have the highest knowledge score compared with others.

**Table 3:** Correlations between various socio-demographic characteristics of the surveyed participants and their knowledge regarding cervical cancer risk factors and HPV vaccine.

**Variable**	**Categories**	**Knowledge score**		**p value**
		**Mean**	**SD**	
**Age in years (n=422)**	Less than 15	7.33	3.786	0.088
	15–25	7.62	2.504	
	26–35	7.10	2.011	
	36–45	7.02	2.345	
	More than 45	6.74	2.141	
**Marital status (n=422)**	Single	7.41	2.372	0.849
	Married	7.14	2.257	
	Divorced	7.15	2.304	
	Separated	7.33	2.582	
	Widowed	7.17	2.483	
**Education (n=422)**	Not educated	10.00	–	0.256
	High school or less	7.64	2.611	
	Bachelor's degree	7.17	2.236	
	Postgraduate degree	7.23	2.201	
**Occupation (n=422)**	Healthcare employee	7.57	2.255	< 0.001
	Non-healthcare employee	6.72	2.036	
	Unemployed	6.96	2.007	
	Student in health specialties	9.30	2.412	
	Student in non-health specialties	6.58	2.242	
**Husband’s education (n=195)**	High school or less	7.33	1.957	0.584
	Bachelor's degree	7.09	2.373	
	Postgraduate degree	6.64	2.767	
**Husband’s occupation (n=195)**	Healthcare worker	6.57	2.574	0.148
	Non-healthcare worker	7.24	2.193	
**Total perceived family monthly income in SAR (n=422)**	Less than 5,000	7.26	2.419	0.888
	5,000–10,000	7.34	2.037	
	More than 10,000	7.22	2.491	

## 3 Discussion

This study was carried out to scrutinize the knowledge about cervical cancer risk factors and HPV vaccine among 422 Saudi women of childbearing age. Out of a total of 14 points, the mean knowledge score for all surveyed participants was about mid-way at seven points. More than three-quarters of the surveyed participants correctly identified several risk factors of cervical cancer. However, only very marginal proportions of the surveyed participants (<30%) correctly answered questions about HPV vaccine in terms of the targeted age group and regimen schedule. Additionally, less than 5% of the participants received HPV vaccine. Close to 40% of the participants declined to receive the HPV vaccine, due to reasons pertaining to lack of awareness and anxiety from injection and adverse events. Among several socio-demographic factors, only occupation was significantly associated with knowledge score, with students in health specialties exhibiting the highest knowledge score compared with others.

In our study, close to 83% of participants correctly identified HPV infection as a risk factor for cervical cancer. This percentage was far higher compared with other local studies in Saudi Arabia by Al Ghamdi (64%) [14], Al-Darwish et al. (53%) [26], and Sait (14%) [22]. The poor awareness about HPV infection as a key underlying causative agent for cervical cancer has been collectively documented among various Arabic countries, including United Arab Emirates, Jordan, Qatar, and Iraq [11]. This deprived knowledge about HPV vaccine is also extended to other global countries, including high-income ones such as China [27], Spain [28], and United Kingdom [29].

Our findings pinpointed several alarming concerns about the status of HPV vaccine in Saudi Arabia. Most notably, around 36.3% of the surveyed participants reported not hearing about HPV vaccine and close to half of the surveyed participants claimed to understand its purpose. All in all, these findings suggest poor public awareness about the HPV vaccine and its central goal to prevent cervical cancer and other related infections. Besides, these data highlight a critical mismatch between the participants’ self-reported understanding of the purpose of the HPV vaccine and paradoxically not receiving it. Surprisingly, only around 4% of the surveyed participants had actually received the HPV vaccine, and this figure is very frightening and warrants an immediate investigation.

In the present investigation, the three most common reasons to reject receiving HPV vaccine encompassed lack of awareness and anxiety from injection and adverse events. These reasons were mirrored in a multitude of studies including Jordan [30], Italy [31], United States of America [32], and various local studies in Saudi Arabia [16], [33], [34].

A wide array of socio-demographic characteristics can substantially impact the acceptability and likelihood of understanding the cervical cancer risk factors and receiving the HPV vaccine. Within these lines, our investigation depicted a positive correlation between occupation and knowledge score, in which students in health specialties displayed the highest knowledge scores compared with others. Indeed, education contributes a major role in understanding HPV vaccine and several lines of investigations documented that individuals who have higher education or those who matriculate in health science colleges appeared to harbor better knowledge and perception of HPV vaccination [13].

All in all, assessing the level of knowledge about cervical cancer risk factors and HPV vaccine is vital as it reflects the population awareness and emphasizes the perceived barriers to accepting HPV vaccine. Afterward, an evidence-based data can be generated that can be rationally utilized to identify the strengths and further reinforce them, as well as to pinpoint the weaknesses and devise deliberated efforts to rectify them.

Our results call for some recommendations that should be implemented regionally in Saudi Arabia. Such recommendations comprise: (i) focused education of women about HPV vaccine to prevent cervical cancer and related infections, (ii) intensive education of physicians and other healthcare providers (for example, nurses) about encouraging HPV vaccine to patients by highlighting its efficacy, safety, and benefits using simplified evidence-based data to support the claims, (iii) formal incorporation of HPV vaccination as a routine practice during outpatient clinic visits, (iv) mandating HPV vaccine as a prerequisite prior to enrolling in school, job, or travel, (v) offering financial and non-financial incentives to those who receive HPV vaccine, and (vi) providing public awareness via various vehicles, such as malls, social media, radio stations, television programs, community campaigns, and social events.

Our study has several strengths that ought to be emphasized. To the best of our knowledge, we reported the first-ever focused study on the knowledge of cervical cancer risk factors and HPV vaccine among Saudi women of childbearing age, and hence our results enrich literature by contributing data from an important geographic region in Saudi Arabia. Moreover, we examined the knowledge of the surveyed participants using a questionnaire that was devised from three published reports and the formulated survey successfully passed validity and reliability testing, hence rendering the presented results more trustworthy. In addition, we quantitively examined correlations between various socio-demographic characteristics of the surveyed participants and knowledge score of cervical cancer risk factors and HPV vaccine. Such correlations favorably permit for recognition of social determinants that can associate with negative attitudes toward cervical cancer prevention.

Nonetheless, our study is not without shortcomings that should be acknowledged. Such shortcomings comprise the relatively small sample size, which could have impacted the power of the deduced conclusions. Further shortcomings include the subjective, self-reporting nature of the collected data; hence the results may be liable to underestimation or overestimation by the participants. The cross-sectional methodological design of the study severely limits the capacity to solidly establish causal inferences among the examined associated factors. Lastly, the surveyed participants were not provided with open-ended questions to express concerns that were not initially addressed by the survey.

Future research includes conducting a large-sized study to gauge the level of awareness about cervical cancer prevention and HPV vaccine acceptability in order to provide better powered conclusions. Additional prospective investigation includes examining the qualitative perceptions of women toward HPV vaccine using semi-focused structured interviews to gain insights into perspectives that would otherwise not be captured via self-reporting surveys. Another interesting study will be to explore the relationship between personal beliefs and acceptability of receiving HPV vaccine.

## 3 Conclusion

This study examined the knowledge about cervical cancer risk factors and HPV vaccine among 422 Saudi women of childbearing age. Most participants displayed good knowledge about cervical cancer risk factors, but not about HPV vaccine. Very alarmingly, less than 5% of the participants received HPV vaccine and close to 40% of them declined to receive the HPV vaccine, due to reasons pertaining to lack of awareness and anxiety from injection and adverse events. Among several socio-demographic factors, only occupation was significantly associated with knowledge score, with students in health specialties exhibiting the highest knowledge score compared with others. The findings of the present investigation should be interpreted with caution in view of the associated shortcomings. Mechanisms to increase public awareness about HPV vaccine and its acceptance by women are recommended.

## Ethical considerations

The study protocol was approved by the Institutional Review Board (IRB) vide Letter No. HAPO-02-K-012-2022-11-1227 dated 02/11 2022 from Umm Al-Qura University, Makkah, Saudi Arabia.

## Declaration of Competing Interest

The authors declare that they have no known competing financial interests or personal relationships that could have appeared to influence the work reported in this paper.

## Data Availability

Data will be made available on request.
